# Three-month follow-up after severe COVID-19 infection: are chest CT results associated with respiratory outcomes and respiratory recovery in COVID-19 patients?

**DOI:** 10.1186/s12890-023-02370-2

**Published:** 2023-03-07

**Authors:** Marlou THF Janssen, Mark GH Thijssen, Jasenko Krdzalic, Michiel HM Gronenschild, Sofia Ramiro, César Magro-Checa, Robert BM Landewé, Rémy LM Mostard

**Affiliations:** 1 Department of Pulmonology, Zuyderland Medical Centre, Henri Dunantstraat 5, 6419 PC Heerlen, Limburg, The Netherlands; 2 Department of Radiology, Zuyderland Medical Centre, Heerlen, Limburg, The Netherlands; 3 Department of Rheumatology, Zuyderland Medical Centre, Heerlen, Limburg, The Netherlands; 4 Department of Rheumatology, Leiden University Medical Centre, Leiden, Zuid-Holland, The Netherlands; 5 Department of Clinical Immunology & Rheumatology, Amsterdam Rheumatology Centre, Amsterdam, The Netherlands; 6 Department of Pulmonology, Maastricht University Medical Centre+, Maastricht, Limburg, The Netherlands

**Keywords:** COVID-19, Hyperinflammation, CT severity score

## Abstract

**Background:**

CT Severity Score (CT-SS) can be used to assess the extent of severe coronavirus disease 19 (COVID-19) pneumonia. Follow-up CT-SS in patients surviving COVID-19-associated hyperinflammation and its correlation with respiratory parameters remains unknown. This study aims to assess the association between CT-SS and respiratory outcomes, both in hospital and at three months after hospitalization.

**Methods:**

Patients from the COVID-19 High-intensity Immunosuppression in Cytokine storm Syndrome (CHIC) study surviving hospitalization due to COVID-19 associated hyperinflammation were invited for follow-up assessment at three months after hospitalization. Results of CT-SS three months after hospitalization were compared with CT-SS at hospital admission. CT-SS at admission and at 3-months were correlated with respiratory status during hospitalization and with patient reported outcomes as well as pulmonary- and exercise function tests at 3-months after hospitalization.

**Results:**

A total of 113 patients were included. Mean CT-SS decreased by 40.4% (SD 27.6) in three months (P < 0.001). CT-SS during hospitalization was higher in patients requiring more oxygen (P < 0.001). CT-SS at 3-months was higher in patients with more dyspnoea (CT-SS 8.31 (3.98) in patients with modified Medical Council Dyspnoea scale (mMRC) 0–2 vs. 11.03 (4.47) in those with mMRC 3–4). CT-SS at 3-months was also higher in patients with a more impaired pulmonary function (7.4 (3.6) in patients with diffusing capacity for carbon monoxide (DLCO) > 80%pred vs. 14.3 (3.2) in those with DLCO < 40%pred, P = 0.002).

**Conclusion:**

Patients surviving hospitalization for COVID-19-associated hyperinflammation with higher CT-SS have worse respiratory outcome, both in-hospital and at 3-months after hospitalization. Strict monitoring of patients with high CT-SS is therefore warranted.

## Background

Coronavirus disease 19 (COVID-19) is a heterogeneous [1]. Chest computed tomography (CT) scan can be helpful to diagnose a COVID-19 pneumonia. Chest CT can be of added value to differentiate between high or low probability of the infection, as reported in the CO-RADS [2]. Furthermore, chest CT can be used to visualize the extent of the disease, as reported in the CT Severity Score (CT-SS)[3, 4]. Previous research has shown that CT-SS in the acute phase of the disease correlates with disease burden and mortality risk [3, 5–9]. Few studies examined the medium- to long-term radiological abnormalities after COVID-19 and the possible correlation with clinical symptoms during admission. Patients surviving severe COVID-19 have higher prevalence of residual lesions on the chest CT scan in comparison with patients surviving mild COVID-19 [10, 11]. Furthermore, one-third of patients had pulmonary fibrotic-like changes six months after severe COVID-19, and these changes were associated with a higher CT-SS at hospital [12]. Nevertheless, none of the studies assessed the relationship between the severity of pulmonary involvement and respiratory outcomes, i.e. the correlation of CT-SS with pulmonary- and exercise functions tests.

A proportion of COVID-19 patients develop a hyperinflammatory state, which leads to an increased risk of respiratory insufficiency and thromboembolic events, and therefore to a higher mortality [13].

The use of immunosuppressive therapy in this state of severe COVID-19 accelerates respiratory recovery and reduces mortality [14–16], therefore glucocorticoids and tocilizumab are recommended in this phase [17]. None of the studies published so far focused on patients with COVID-19-associated hyperinflammation or assessed the influence of glucocorticoids and tocilizumab on the resolution of radiological findings.

The primary objective of this study was to investigate the association between CT-SS and respiratory outcomes at both the moment of hospitalization and at three months after hospitalization.

The secondary objective was to investigate whether intensive short-term immunosuppressive therapy for COVID-19 was associated with a lower CT-SS at three months after hospitalization compared with patients who did not receive this immunosuppressive therapy.

## Methods

### Study design and subjects

This prospective cohort study included patients who survived hospitalization for COVID-19-associated hyperinflammation. Between March and May 2020, patients hospitalized for COVID-19-associated hyperinflammation in the Zuyderland Medical Centre (ZMC), a large teaching hospital in the Netherlands, were included in the COVID High-intensity Immunosuppression in Cytokine storm syndrome (CHIC) study [14]. COVID-19-associated hyperinflammation was defined according to a set of criteria: oxygen saturation at rest ≤ 94% or tachypnoea (> 30/min); at least two out of three biomarker criteria: CRP > 100 mg/L, serum ferritin > 900 µg/L at one occasion or a twofold increase of the level at admission within 48 h and D-dimer level > 1500 µg/ [14]. From March 1st to April 1st 2020, patients were treated with standard of care (control group), consisting of oxygen support, antibiotics, chloroquine and anticoagulation. After April 1st, patients were treated according to the CHIC protocol, which was added to standard of care (treated group). This protocol included two steps: (1) intravenous methylprednisolone 250 mg on day 1, followed by methylprednisolone 80 mg intravenously on days 2–5, and an option for a two-day extension; (2) addition of tocilizumab (single dose, 8 mg/kg body weight intravenous, max 800 mg) in case of lack of improvement or worsening in respiratory status 48 h after starting with methylprednisolone. Results confirming the benefits of this therapeutical strategy during the acute setting of COVID-19-associated hyperinflammation have been published [14]. All survivors of this study were invited for an ambulatory follow-up visit at the Pulmonology department of ZMC. Patients were excluded if they were unable to visit the outpatient clinic.

Approval was obtained by the Medical Ethical Committee (METC) and the Board of ZMC, the Netherlands (number METCZ20200126). All patients provided written informed consent for the use of their data for this study.

### Procedures and data collection

Patients were invited to the outpatient clinic at three months after hospital discharge. Patients were assessed by a pulmonologist and pulmonary- and exercise function tests and a chest CT were performed. Furthermore, patients completed questionnaires.

#### Chest CT protocol and evaluation

Chest CT scans were performed at the same time in each patient, namely during hospitalization at the moment of fulfillment of the criteria used for defining hyperinflammation and at three months after hospitalization. Chest CT was performed on either a 64-slice CT scanner (Philips Incisive) or on a 64-slice dual source scanner (Siemens Somatom Definition Flash). Scanning parameters were: collimation 64 × 0.625 or 0.6 mm,120 kVp, 667 max mA or 404 max mA, pitch 1.0 or 1.2, and matrix size 512 × 512. CT images were reconstructed with a lung kernel in the transverse plane with 1.0-mm slice thickness and 1.0-mm increment. Images were also reconstructed in axial, coronal, and sagittal planes with 3.0-mm slice thickness. All images were stored in the local picture archiving and communication system (Sectra IDS7 PACS). Severity of pulmonary involvement was assessed using CT-SS [3]. Per each of the five pulmonary lobes, the anatomic parenchymal involvement was calculated: 0, no involvement; 1, < 5% involvement; 2, 2–25% involvement; 3, 26–50% involvement; 4, 51–75% involvement; 5, > 75% involvement. CT-SS was the result of the sum of each individual lobar score (total score 0–25) [3]. For the purpose of this study, CT-SS was retrospectively scored by one experienced thoracic radiologist and one experienced pulmonologist, they scored the CT scans independently from each other and with known chronological order. They were blinded for the patients’ clinical information, including the therapy received during hospitalization. The scores of both readers were assessed separately and subsequently averaged. Reliability of the CT-SS between both readers was assessed using the intraclass correlation coefficient (ICC).

#### Patient reported outcomes

Patients completed the modified Medical Research Council dyspnoea scale (mMRC) to assess perceived functional limitations of breathlessness, the Hospital Anxiety and Depression Scale (HADS) questionnaire to detect states of anxiety and depression and the EuroQol 5-dimensions 5-levels (EQ-5D-5 L) questionnaire for measuring quality of [18–21].

#### Pulmonary- and exercise function tests

Forced vital capacity (FVC), total lung capacity (TLC) and diffusing capacity for carbon monoxide (DLCO) were tested with a pneumotachograph (Vyaire Medical, Jaeger, Würzburg, Germany). DLCO was measured using the single-breath carbon monoxide uptake. Values were calculated using Global Lung Function Initiative network references. A six-minute walk test (6MWT) with pulse oximetry was performed to objectively evaluate functional exercise. Pulmonary function tests and 6MWT were performed according to the European Respiratory Society and American Thoracic Society guidelines and values were expressed as a percentage of predicted values [22–25].

### Statistical analysis

Reliability of the CT-SS, for both status scores at baseline and 3 months as well as change score (baseline minus 3-month score) was assessed using the intraclass correlation coefficient (ICC) with two-way mixed model and absolute agreement.

Descriptive statistics, namely mean and range values, were used for all outcomes. Differences in baseline characteristics between treatment groups were computed with a Mann-Whitney U test or a χ^2^/Fisher’s exact test. CT-SS between hospital CT scans and follow-up CT scans were compared using a paired samples t-test.

An independent samples t-test or Mann-Whitney U test was used for detecting differences in CT-SS between two different groups and one-way ANOVA or Kruskal Wallis was used for detecting differences in CT-SS across more than two groups. ANCOVA was used to correct for baseline values.

A p-value ≤ 0.05 was considered statistically significant. Data were analysed with the statistical package SPSS Statistics version 26.

## Results

**Fig. 1:**
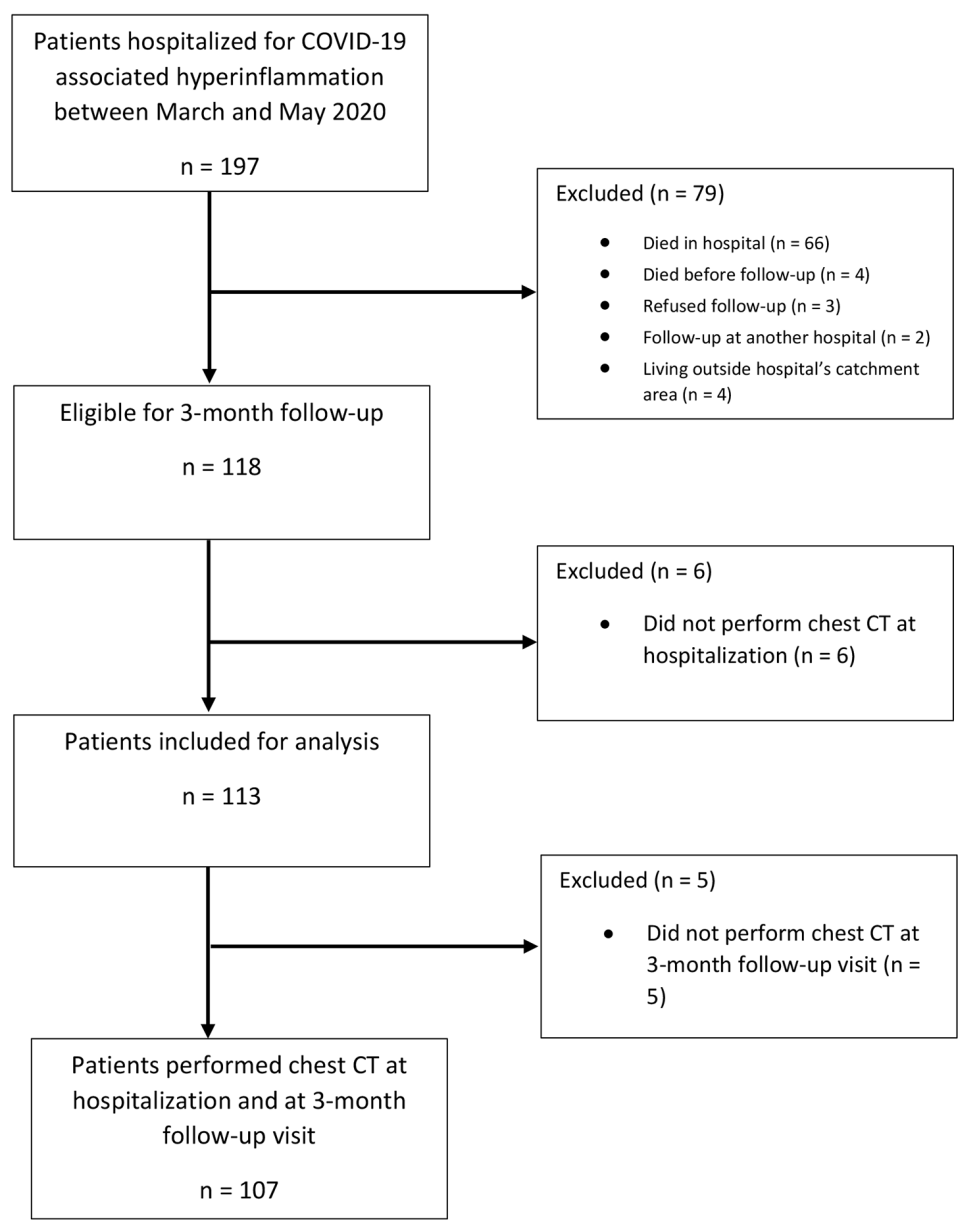
Flowchart of included patients

One hundred thirteen patients were included in this follow-up, having their CT scan at three months after hospitalization. Of all 113 patients, 107 performed a chest CT during hospitalization and after three months. Patient inclusion is shown in Fig. 1 and baseline characteristics are presented in Table 1. The mean age was 63 (SD 11) years and 82% of the patients were male. Hypertension was the most common comorbidity. At the moment of fulfillment of the criteria for hyperinflammation, 46% of the patients in the control group needed an oxymask/non-rebreathing mask, high flow nasal canula (HFNC) or mechanical ventilation, versus 45% in the treated group, though more patients in the control group were mechanically ventilated (16% vs. 1.6%).

The ICC between both readers was 0.87 (95% CI 0.50–0.94) and 0.89 (95% CI 0.79–0.93) for the chest CT performed during hospitalization and at the three-month follow-up visit, respectively. The ICC for the change in CT-SS between hospitalization and three-month follow-up was 0.91 (95% CI 0.87–0.94).

**Fig. 2:**
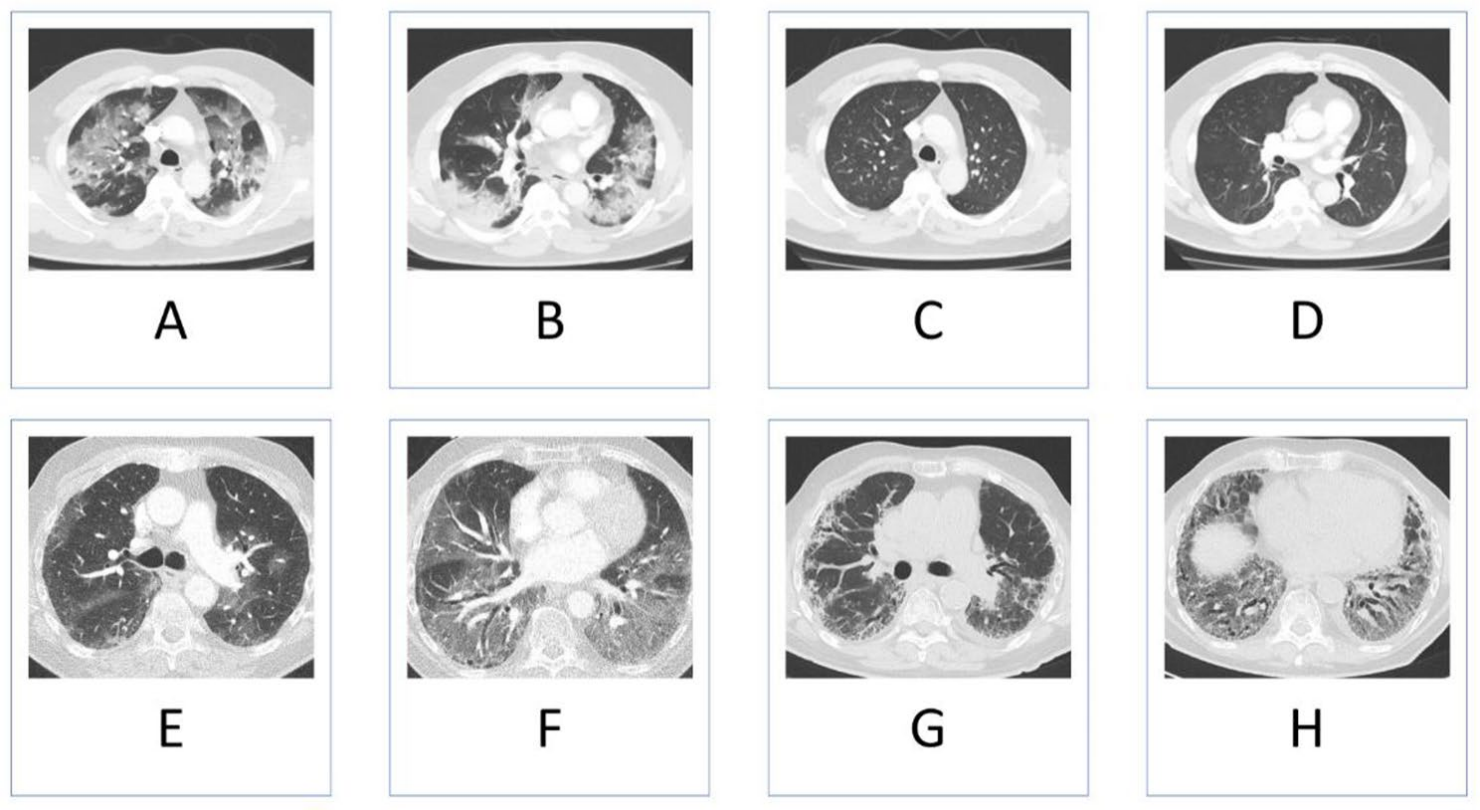
Demonstrative CT scan images. 68-year old man with CT-SS of 19 at the acute phase of COVID-19 (A and B) and CT-SS of 0 at the three-month follow-up visit (C and D). Pulmonary function tests and six minute walk test at the three month follow-up visit were unremarkable. 86-year old patient with CT-SS of 16 at the acute phase of COVID-19 (E and F) and CT-SS of 13 at the three month follow-up visit (G and H). Pulmonary function test at the three month follow-up visit showed a TLC (Total Lung Capacity) of 58% predicted and a DLCO (Diffusing Capacity for Carbon Monoxide) of 29% predicted. The six minute walk test was not feasible due to the clinical condition

At baseline, i.e. during hospitalization, the mean CT-SS was 15.2 (SD 3.8). At the three-month follow-up visit, the mean CT-SS was 8.8 (SD 4.2). The mean CT-SS decreased significantly by 6.4 (SD 4.6), which corresponds to a 40.4% (SD 27.6) decrease in three months (P < 0.001). Figure 2 shows demonstrative CT scan images of two patients with pulmonary abnormalities at hospitalization and at the three-month follow-up visit.

**Table 1:** Baseline demographic and clinical characteristics

	Total (N = 113)	Control group (N = 50)	Treated group (N = 63)	P-value*
Age (years)	63 (11)	62 (9.7)	64 (11.9)	0.367
Male gender	93 (82.3%)	43 (86%)	50 (79.4%)	0.359
BMI (kg/m^2^)	29.1 (5.2)	30.6 (5.62)	27.9 (4.52)	0.085
Smoking				0.025
- Never smoker	36 (31.9%)	21 (42%)	15 (23.8%)	
- Ex-smoker	65 (57.5%)	28 (56%)	37 (58.7%)	
- Current smoker	3 (2.7%)	0	3 (4.8%)	
Hypertension	31 (27.4%)	18 (36%)	13 (20.6%)	0.069
Diabetes Mellitus	23 (20.4%)	14 (28%)	9 (14.3%)	0.072
COPD	13 (11.5%)	4 (8%)	9 (14.3%)	0.298
Asthma	8 (7.1%)	3 (6%)	5 (7.9%)	0.494
Malignancy	1 (0.9%)	0	1 (1.6%)	0.558
Hematologic malignancy	2 (1.8%)	0	2 (3.2%)	0.309
Cardiovascular disease	17 (15%)	6 (12%)	11 (17.5%)	0.420
Heart failure	1 (0.9%)	0	1 (1.6%)	0.558
Arrhythmia	11 (9.7%)	4 (8%)	7 (11.1%)	0.412
Chronic kidney disease	3 (2.7%)	2 (4%)	1 (1.6%)	0.413
Cerebrovascular disease	7 (6.2%)	5 (10%)	2 (3.2%)	0.136
Peripheral vascular disease	6 (5.3%)	3 (6%)	3 (4.8%)	0.545
Auto-immune disease	13 (11.5%)	7 (14%)	6 (9.5%)	0.459
Charlson Comorbidity Index	0.84 (1.11)	0.94 (1.13)	0.75 (1.10)	0.295
Oxygen support at baseline†				0.029
- Nasal oxygen	62 (54.9%)	27 (54%)	35 (55.6%)	
- Oxymask/NRM	28 (24.8%)	11 (22%)	17 (27%)	
- High flow nasal cannula	14 (12.4%)	4 (8%)	10 (15.9%)	
- Mechanical ventilation	9 (8.0%)	8 (16%)	1 (1.6%)	
WHO score baseline†**				0.012
- 4: hospitalisation, requiring oxygen	90 (79.6%)	37 (76%)	52 (82.5%)	
- 5: hospitalisation, requiring high-flow nasal oxygen therapy or non-invasive ventilation	14 (12.4%)	4 (8%)	10 (15.9%)	
- 6: hospitalisation, requiring ECMO, invasive mechanical ventilation or both	9 (8.0%)	8 (16%)	1 (1.6%)	

Data are presented as N (%) or mean (SD)

* Calculated with Mann-Whitney U test of χ^2^/Fisher’s exact test, as appropriate

†Baseline is at the day on which patients fulfilled the criteria for hyperinflammation during hospitalisation

** WHO score: The WHO score consists of 7 stages which are: (1) non-hospitalized, able to resume normal activities; (2) non-hospitalized, but unable to resume normal activities; (3) hospitalized, not requiring oxygen therapy; (4) hospitalized, requiring additional oxygen therapy; (5) hospitalized, requiring high-flow nasal oxygen therapy, non-invasive mechanical ventilation or both; (6) hospitalized, requiring extracorporeal membrane oxygenation, mechanical ventilation or both; and (7) death. The scale was used from 2 to 7 for this study

BMI, body mass index; COPD, chronic obstructive pulmonary disease; NRM, non-rebreathing mask; WHO, World Health Organisation; ECMO, extracorporeal membrane oxygenation

### CT-SS during hospitalization and relationship with respiratory outcomes

Mean CT-SS was significantly higher in patients requiring more oxygen and in patients requiring more oxygen suppletion anytime during hospitalization (18.3 (3.7) for patients requiring mechanical ventilation vs. 13.6 (3.1) for patients requiring nasal oxygen, with a decreasing gradient between both groups, P < 0.001) (Table 2).

**Table 2:** CT-severity score and oxygen suppletion during hospitalization

	Range CT-SS (minimum-maximum)	Mean CT-SS Mean (SD)	P-value *
WHO score baseline†			P = 0.022
- 4: hospitalisation, requiring oxygen (N = 87)	6.5–24.0	14.7 (3.7)	
- 5: hospitalisation, requiring high-flow nasal oxygen therapy or non-invasive ventilation (N = 14)	11.0–22.5	16.9 (3.4)	
- 6: hospitalisation, requiring ECMO, invasive mechanical ventilation or both (N = 7)	12.0–23.0	17.8 (4.0)	
Maximum oxygen suppletion during the whole hospitalization			P < 0.001
- Nasal oxygen (N = 42)	6.5–22.5	13.6 (3.1)	
- Oxymask/NRM (N = 23)	9.0–23.0	14.8 (3.7)	
- High flow nasal cannula (N = 19)	8.0–19.0	15.32 (3.2)	
- Mechanical ventilation (N = 24)	11.5–24.0	18.23 (3.7)	

†WHO score: The WHO score consists of 7 stages which are: (1) non-hospitalized, able to resume normal activities; (2) non-hospitalized, but unable to resume normal activities; (3) hospitalized, not requiring oxygen therapy; (4) hospitalized, requiring additional oxygen therapy; (5) hospitalized, requiring high-flow nasal oxygen therapy, non-invasive mechanical ventilation or both; (6) hospitalized, requiring extracorporeal membrane oxygenation, mechanical ventilation or both; and (7) death. The scale was used from 2 to 7 for this study

†Baseline is at the day on which patients fulfilled the criteria for hyperinflammation during hospitalisation

*Calculated with one-way ANOVA or Kruskal Wallis, as appropriate.

CT-SS, CT severity score; WHO, World Health Organisation; ECMO, extracorporeal membrane oxygenation; NRM, non-rebreathing mask

Patients with a higher CT-SS during hospitalization had a more impaired pulmonary function three months after hospitalization (16.6 (4.0) for patients with a TLC < 80%pred vs. 14.8 (3.7) for patients with a TLC ≥ 80%pred, P = 0.041, and 17.4 (4.2) for patients with a DLCO < 40%pred vs. 14.0 (3.9) for patients with a DLCO > 80%pred, P = 0.026). For DLCO, higher CT-SS was associated with a lower DLCO. Patients with a more pronounced desaturation during 6MWT did not have a significantly higher CT-SS during hospitalization, however the minimum CT-SS was higher in the group with a more pronounced desaturation (Table 3).

**Table 3:** CT-severity score at hospitalization and at 3-month follow-up visit; and pulmonary and exercise function tests at three-month follow-up visit

	CT-SS at hospitalization			CT-SS at 3-month follow-up visit		
	Range CT-SS (minimum-maximum)	Mean CT-SS Mean (SD)	P-value*	Range CT-SS (minimum-maximum)	Mean CT-SS Mean (SD)	P-value*
**Pulmonary function tests**						
**TLC**			P = 0.041			P < 0.001
≥ 80% pred (n = 84 at hospitalization) (n = 87 at 3-month follow-up)	6.5–23.5	14.8 (3.7)		2.5–19.0	7.8 (3.8)	
< 80% pred (n = 22 at hospitalization) (n = 24 at 3-month follow-up)	8.5–24.0	16.6 (4.0)		2.5–18.5	11.2 (3.9)	
**DLCO**			P = 0.026			P = 0.002
> 80% pred (n = 42 at hospitalization) (n = 47 at 3-month follow-up)	6.5–19.5	14.0 (3.9)		2.5–17.5	7.4 (3.6)	
60–80% pred (n = 37 at hospitalization) (n = 36 at 3-month follow-up)	8.0–23.0	15.4 (3.8)		3.5–17.0	8.7 (3.5)	
40–60% pred (n = 22 at hospitalization) (n = 22 at 3-month follow-up)	8.0–24.0	16.6 (4.7)		3.0–18.5	9.4 (4.3)	
< 40% pred (n = 5 at hospitalization) (n = 6 at 3-month follow-up)	12.5–22.0	17.4 (4.2)		10.5–19.0	14.3 (3.2)	
**Exercise function test**			P = 0.284			P = 0.098
6MWT saturation ≥ 90% (n = 67 at hospitalization) (n = 71 at 3-month follow-up)	6.5–23.5	14.8 (3.6)		2.5–17.5	8.1 (3.8)	
6MWT saturation 85–89 (n = 20 at hospitalization) (n = 20 at 3-month follow-up)	8.0–24.0	16.2 (4.1)		3.5–18.5	10.3 (4.5)	
6MWT saturation ≤ 84 (n = 5 at hospitalization) (n = 5 at 3-month follow-up)	10.0–18.0	14.2 (3.5)		3.5–9.0	6.6 (2.6)	

*Calculated with independent samples T-test or Mann-Whitney U, as appropriate and with one-way ANOVA or Kruskal-Wallis, as appropriate.

CT-SS, CT severity score; TLC, Total Lung Capacity; DLCO Diffusing Capacity for Carbon Monoxide); 6MWT, six Minute Walk Test

### CT-SS at three-month follow-up and relationship with respiratory outcomes

Mean CT-SS at three months was significantly higher in patients who required more oxygen during hospitalization, with an increasing gradient with increasing oxygen suppletion (p = 0.007) (Table 4).

Patients with a more impaired pulmonary function had a significantly higher mean CT-SS, with an increasing gradient of CT-SS with a decreasing DLCO (P < 0.001 and P = 0.002 for TLC and DLCO, respectively). Patients with a more pronounced desaturation during 6MWT did not have a significantly higher CT-SS at that time (P = 0.098) (Table 3).

Higher mMRC score at the three-month follow-up visit was associated with higher CT-SS at three months. Mean CT-SS was 8.31 (SD 3.98) in patients with a reported mMRC of 0–2 and 11.03 (SD 4.47) in patients with a reported mMRC of 3–4 (P = 0.020).

HADS and EQ-5D questionnaire results did not correlate with CT-SS (HADS anxiety Spearman’s rho

-0.104, HADS depression Spearman’s -0.120, EQ-5D VAS Spearman’s rho 0.141).

**Table 4:** CT-severity score at three months and oxygen suppletion during hospitalization

	Range CT-SS (minimum-maximum)	Mean CT-SS Mean (SD)	P-value *
WHO score baseline†			P = 0.194
- 4: hospitalisation, requiring oxygen (N = 90)	2.5–20.0	8.3 (4.0)	
- 5: hospitalisation, requiring high-flow nasal oxygen therapy or non-invasive ventilation (N = 14)	3.5–18.5	10.4 (5.0)	
- 6: hospitalisation, requiring ECMO, invasive mechanical ventilation or both (N = 9)	4.0–12.5	9.4 (2.9)	
Maximum oxygen suppletion during the whole hospitalization			P = 0.007
- Nasal oxygen (N = 44)	2.5–14.5	7.1 (3.2)	
- Oxymask/NRM (N = 23)	3.5–19.0	8.8 (4.7)	
- High flow nasal cannula (N = 19)	3.5–20.0	9.2 (4.6)	
- Mechanical ventilation (N = 27)	3.5–18.5	10.6 (4.0)	

†WHO score: The WHO score consists of 7 stages which are: (1) non-hospitalized, able to resume normal activities; (2) non-hospitalized, but unable to resume normal activities; (3) hospitalized, not requiring oxygen therapy; (4) hospitalized, requiring additional oxygen therapy; (5) hospitalized, requiring high-flow nasal oxygen therapy, non-invasive mechanical ventilation or both; (6) hospitalized, requiring extracorporeal membrane oxygenation, mechanical ventilation or both; and (7) death. The scale was used from 2 to 7 for this study

†Baseline is at the day on which patients fulfilled the criteria for hyperinflammation during hospitalisation

*Calculated with one-way ANOVA or Kruskal Wallis, as appropriate

CT-SS, CT severity score; WHO, World Health Organisation; ECMO, extracorporeal membrane oxygenation; NRM, non-rebreathing mask

### Differences in CT-SS between treated and control groups

During hospitalization, CT-SS of the treated group (methylprednisolone with or without tocilizumab) was 14.60 (SD 3.46) and CT-SS of the control group (i.e. standard care / no immunomodulatory therapy) was 16.10 (SD 4.09), P = 0.042. At three-month follow-up, CT-SS was 9.47 (SD 4.25) and 7.91 (SD 3.86) for the treated and control group, respectively (P = 0.055). Corrected for baseline differences in CT-SS, gender, age and important comorbidities (hypertension, diabetes mellitus, COPD, cardiovascular disease, cerebrovascular disease, auto-immune disease), the difference between the groups at the three-month follow-up visit was statistically significant (p = 0.029).

The decrease in CT-SS between hospitalization and the three month follow-up visit was lower for the treatment group in comparison with the control group (5.13 (SD 4.56) versus 8.19 (SD 4.16), P = 0.001). Corrected for the same variables as previously mentioned, the difference between the treatment and control groups remained statistically significant (p = 0.004).

## Discussion

This prospective cohort study shows that chest CT abnormalities decrease significantly in three months after surviving hospitalization for COVID-19-associated hyperinflammation. Patients with a higher CT-SS (in hospital and at three months) required higher maximum oxygen suppletion during hospitalization, had significant more impaired TLC and DLCO measurements and experienced more dyspnea at three months after hospitalization.

Based on these results, higher CT-SS may be used as an indicator for worse respiratory status during hospitalization, more dyspnoea and pulmonary function impairments after three months. CT-SS may therefore be used for evaluating individual burden of disease and physicians should be aware of a possible worse outcome in patients with higher CT-SS.

In the acute phase of the pandemic (especially first quarter 2020) it was common practice to use a chest CT for screening for COVID-19, mainly because of lack of polymerase chain reaction (PCR) tests. Nowadays, chest CT is not used for screening for COVID-19. The data from all performed CT-scans is helpful for follow-up though.

To our knowledge, this is the first study that compares systematically performed chest CT during the acute phase of the disease with medium-term follow-up chest CT in a homogeneous population of patients who survived COVID-19-associated hyperinflammation.

Previous studies found cross-sectional associations between higher CT-SS and more severe disease during hospitalization [3, 7, 26, 27], however data on follow-up CT-SS were not included. Other studies found that more radiological abnormalities at six and 12 months after hospitalization were associated with more oxygen requirement during hospitalization [11, 28], which is consistent with our findings. However, one study was performed retrospectively and included only 48 patients [11], the other study excluded patients with severe disease and comorbidities [28]. Additionally, in contrast to the current study, none of the previous studies performed systematically a chest CT at the acute phase of the disease. Furthermore, none of the studies assessed the relationship between CT-SS during and after hospitalization with patient reported-, pulmonary- and exercise test outcomes. CT-SS at the moment of COVID-19 differed between studies; however, one study including only patients with severe COVID-19 also found a mean CT-SS of 15 [12].

As expected, CT-SS was higher in patients requiring more oxygen. SARS-CoV-2 mainly affects the pulmonary alveoli and the surrounding vascular components. Interstitial inflammatory infiltrates and edema lead to hypoxemia. More infiltrates and edema will lead to more severe respiratory [1]. Over time, alveolar infiltrates and edema will diminish. Persisting radiological abnormalities, as well as an impaired TLC and DLCO may be the result of incomplete resolution of this damage. However, it may be possible that part of the patients had pre-existing (fibrotic) radiological abnormalities or pulmonary function impairments due to other diseases, as we did not have chest CT data or pulmonary function data before onset of COVID-19. Our study showed no higher CT-SS in patients with a more pronounced desaturation during 6MWT, however the minimum CT-SS was higher with increasing desaturation. Due to the small numbers it is hard to draw robust conclusions.

The improvement in CT-SS after three months was higher in the control group than in the treated group, even after correction for baseline differences. Also, CT-SS at 3 months was lower in the control group, compared to the treated group. However, caution must be taken in interpreting these results due to the differences between the groups at baseline. Importantly, in the CHIC study patients were included in a balanced way between treatment and control groups. In the current follow-up study, survivors of the CHIC study were included. The larger number of deaths in the CHIC-study control group, which are most likely the patients with the highest CT-SS, probably resulted in unbalanced baseline groups of the current study concerning this specific analysis [14]. This hampers a conclusion on the effect of treatment on CT-SS at 3 months. Moreover, results of pulmonary- and exercise function tests did not differ between these two groups at the three-month follow-up visit [29]. The beneficial effects of immunosuppression are therefore mainly limited to the first weeks after administation [14].

Strengths of our study are the prospective design, the systematically performed chest CT at the acute phase of the disease, the comprehensive and standardized assessment after three months with chest CT, patient reported outcomes, pulmonary- and exercise tests and the homogeneous population, as all patients included had COVID-19-associated hyperinflammation during hospitalization, which makes this population unique and distinguishes this study from previous ones. The focus on patients with COVID-19-associated hyperinflammation allows to generalize conclusions to this specific population. Also, chest CT during hospitalization was performed at the same time in each patient, namely at the moment of fulfillment of the criteria used for defining hyperinflammation, making them comparable. Furthermore, the excellent inter-rater agreement in CT-SS supports the reproducibility of this radiological scoring system. Limitations of this study include the absence of chest CT data before onset of COVID-19, which is due to the acute and severe nature of the disease. Another limitation is that follow-up chest CT was not available in a small minority of all potentially eligible patients (14/127 eligible patients: 4 control group patients and 10 treatment group patients). Also, the results of the analyses of treatment effect on CT-SS should be interpreted with caution: the extent to which the treatment and the control group are comparable is unknown due to the higher number of deaths in the control group during hospitalization. Furthermore, the relatively small number of patients, especially in subgroups, is a limitation. Besides, no minimal clinical important difference is established in CT-SS, making it hard to draw robust conclusions about absolute differences in CT-SS between groups. Nevertheless, our findings support the hypothesis that patients with more radiological abnormalities have worse respiratory status and outcomes.

## Conclusion

Patients surviving hospitalization for COVID-19-associated hyperinflammation with higher CT-SS have worse respiratory outcome, both in-hospital and at 3-months after hospitalization. Strict monitoring of patients with high CT-SS is therefore warranted. Follow-up studies are needed to evaluate CT-SS and the correlation with clinical outcomes on long-term.

## Data Availability

The datasets used and/or analyzed during the current study are available from the corresponding author on reasonable request.

## List of Abbreviations

COVID-19: Coronavirus disease 19
CT-SS: CT Severity Score
ZMC: Zuyderland Medical Centre
CHIC: COVID High-intensity Immunosuppression in Cytokine storm syndrome
METC: Medical Ethical Committee
mMRC: Modified Medical Research Council dyspnoea scale
HADS: Hospital Anxiety and Depression Scale
EQ-5D-5L: EuroQol 5-dimensions 5-levels
FVC: Forced vital capacity
TLC: Total lung capacity
DLCO: Diffusing capacity for carbon monoxide
6MWT: Six-minute walk test
ICC: Intraclass correlation coefficient
HFNC: High flow nasal canula oxygen therapy
NRM: Non-rebreathing mask
ECMO: Extracorporeal membrane oxygenation
BMI: Body mass index
COPD: Chronic obstructive pulmonary disease
